# 

**Published:** 1897-09-11

**Authors:** 


					T~ THE HOSPITAL. ABSOLUTELY PURE, therefore BEST. Sept. Hth, 1897.
CADBURY'S -a U CADBURY'S
OOCOA 9H8 Bp=5? SbH 'COCOA
1 The standard of highest purity at present
attainable."?Lancet, NO ALKALIES USED.
Q ^ D r % / v ??
our, t;. ;
?UiASQOW ntSTZRH ItiFL&MA.JUT
No- voi. xxii. [KSfpi1] Saturday,
Sept. 11th, 1897.
k ""'NORf.OLKAuaMuR WICH HOSPI.TAL
[SubSoription)Terms|-| pEJC;E TWOPENCE.

				

